# Influence of Menstrual Cycle Estradiol-β-17 Fluctuations on Energy Substrate Utilization-Oxidation during Aerobic, Endurance Exercise

**DOI:** 10.3390/ijerph18137209

**Published:** 2021-07-05

**Authors:** Hannah N. Willett, Kristen J. Koltun, Anthony C. Hackney

**Affiliations:** 1 Department of Exercise & Sport Science, University of North Carolina, Chapel Hill, NC 27599, USA; hwillett@email.unc.edu; 2 Department of Sports Medicine and Nutrition, University of Pittsburgh, Pittsburgh, PA 15203, USA; KJK116@pitt.edu; 3 Department of Nutrition, Gillings school of Global Public Health, University of North Carolina, Chapel Hill, NC 27599, USA

**Keywords:** women, hormones, physical activity, metabolism, eumenorrhea, performance

## Abstract

This study examined the effect of estradiol-β-17 across the menstrual cycle (MC) during aerobic exercise on energy substrate utilization and oxidation. Thirty-two eumenorrheic (age = 22.4 ± 3.8 y (mean ± SD)), physically active women participated in two steady-state running sessions at 65% of VO_2max_, one during the early follicular and one during the luteal phase of the MC. Blood samples were collected at rest before each exercise session and analyzed for Estradiol-β-17 to confirm the MC phase. Carbohydrate (CHO) utilization and oxidation values were significantly lower (*p* < 0.05) in the luteal (utilization: 51.6 ± 16.7%; oxidation: 1.22 ± 0.56 g/min; effect size (ES) = 0.45, 0.27) than follicular phase (utilization: 58.2 ± 15.1%; oxidation: 1.38 ± 0.60 g/min) exercise sessions. Conversely, fat utilization and oxidation values were significantly (*p* < 0.05) higher in the luteal (utilization: 48.4 ± 16.7%; oxidation: 0.49 ± 0.19 g/min; ES = 0.45,0.28) than follicular phase (utilization: 41.8 ± 15.1%; oxidation: 0.41 ± 0.14 g/min). Estradiol-β-17 concentrations were significantly (*p* < 0.01) greater during the luteal (518.5 ± 285.4 pmol/L; ES = 0.75) than follicular phase (243.8 ± 143.2 pmol/L). Results suggest a greater use of fat and reduced amount of CHO usage during the luteal versus follicular phase, directly related to the change in resting estradiol-β-17. Future research should investigate the role these changes may play in female athletic performance.

## 1. Introduction

The number of women engaged in exercise activities for health as well as sporting endeavors has grown exponentially in recent decades [1]. As such, the number of research studies examining female physiological responses to exercise has grown too, although the total amount of work is far less than in men and thus more research is still needed [1]. This last point is warranted as the unique aspects of a woman’s physiology, especially relative to the influence of the menstrual cycle and reproductive hormones, is critical to understand scientifically since many of the findings in male-based studies cannot and should not be directly applied to women (i.e., “*women are not small men*”; Dr. Stacy Sims quote).

Ovulatory, eumenorrheic adult women display large fluctuations in reproductive hormone concentrations throughout a menstrual cycle [2]; low estrogen and progesterone at menses (early follicular phase) with elevations beginning at ovulation and continuing into the luteal phase). Specifically, the steroid hormonal changes in estrogens (primarily estradiol-β-17) and progesterone follow cyclical patterns during a woman’s reproductive years. The depiction of distinct phases within a menstrual cycle—menses, follicular, ovulation, and luteal—have traditionally been used to characterize the underlying reproductive hormone profiles that can be present. For example, the follicular phase is indicated by low concentrations of estrogens and progesterone compared to the luteal phase [2].

While estrogens and progesterone are critical in the reproductive cycle of a woman, they also have additional physiological roles related to aspects of metabolism, hydration, thermoregulation, and behavior [2]. Relative to metabolism, specifically, estradiol-β-17 has been studied extensively due to its role in promoting lipolysis as demonstrated in both animal and human-based research [3].

In light of the above points, we chose to examine the role of menstrual cycle-induced changes in estradiol-β-17 on energy substrate utilization and oxidation in eumenorrheic adult women during aerobic, endurance exercise. We hypothesized that elevations in estradiol-β-17 during the luteal phase of the menstrual cycle would be related to increased fat and decreased carbohydrate use as energy sources during aerobic exercise.

## 2. Materials and Methods

### 2.1. Study Design and Participants

Healthy, physically active, pre-menopausal adult women were recruited for this study. Inclusion criteria stated that participants could not be taking anti-inflammatory medications, oral contraceptives, or other hormone therapies, and were eumenorrheic (at least for 6 months prior to the study). To meet minimum physical activity levels, participants needed to exercise at least 3 times per week for 45–60 min at a moderate to vigorous intensity over the previous year. Potential participants were screened by the investigators for all inclusion criteria and informed consent in accordance with the Helsinki Declaration was obtained. Forty women were recruited into the study (via flyers and online listserves), of which 32 completed all aspects of our protocol which involved three laboratory sessions: (i) an orientation session involving the determination of maximal oxygen uptake (VO_2max_), and two submaximal steady-state running sessions at 65% VO_2max_, (ii) once during the early follicular (days 3–7), and (iii) once during the luteal (days 19–25) phase of the menstrual cycle (which ranged in length from 25 to 33 days). The eight participants excluded were anovulatory (*n* = 2) or self-selected removal from the study (*n* = 6). The design incorporated in this study was repeated measures with every participant serving as their own control.

*Session One—Orientation*. Upon arrival to our laboratory, participants signed an informed consent, completed a medical history questionnaire, and had a physical medical screening including a 12-lead electrocardiogram. Next, their height was measured with a portable stadiometer (Perspectives Enterprises, Portage, MI, USA) and body mass was obtained using a mechanical scale (Detecto, Webb City, MO, USA). These latter values were used to calculate body mass index (BMI). 

VO_2max_ was assessed with an incremental treadmill (Quinton Q65, Bothell, WA, USA) test. For this testing, the participant began with a warm-up at a self-selected pace (~5 min) followed by light stretching. After the warm-up, resting VO_2_ was recorded for three minutes after which the test began and was conducted according to the Bruce treadmill protocol [4]. Respiratory gases were monitored continuously (Parvo Medics TrueMax 2400 Metabolic System, Parvo Medics, Salt Lake City, UT, USA) as was heart rate (HR) using a telemetry system (Polar Electro, Inc., Lake Success, NY, USA). Ratings of perceived exertion (RPE) were determined using Borg’s 20-point scale at the end of each 3-min test stage. Participants were required to meet three of the following criteria for a test to be considered maximal: a plateau of VO_2_ with increases in workload, respiratory exchange ratio (RER) greater than or equal to 1.1, RPE ≥ 18, and/or HR within 10% of predicted maximal HR [4]. 

The VO_2max_ test responses were used to calculate the required treadmill running speed to elicit 65% of VO_2max_ based upon the American College of Sports Medicine guidelines [5]. 

*Sessions Two and Three—Steady-state Aerobic Exercise.* For the submaximal steady-state running sessions, one occurred during the follicular phase and one during the luteal phase of the menstrual cycle in a randomized order. Menstrual cycle phase was determined using the forward counting method as well as urinary ovulation kits [6]. Both steady-state aerobic exercise sessions were completed at the same time of day (i.e., morning or afternoon) depending on the participant’s availability. Participants were told to refrain from strenuous physical activity and complete a food diary for twenty-four hours before the start of each session. The food diary was collected and assessed to ensure adequate consumption of calories and carbohydrates. Prior to beginning each session, participants confirmed they followed all guidelines before proceeding. 

Resting blood samples were collected following ten minutes of supine rest. A 3 mL blood sample was collected using standard venipuncture procedures. Blood samples were placed in a K_2_-EDTA (purple top) Vacutainer^®^ tube and immediately put on ice until later processing to separate plasma (see the following section). 

After blood sampling, the participants completed 5 min of a warm-up (jogging at self-selected pace) and stretching, then began their 60 min treadmill run at a speed to elicit ~65% VO_2max_. The treadmill speed was fixed and not adjusted through these runs and replicated at each of the two sessions. Steady-state respiratory gases were collected at 30 min and 60 min of the runs to determine VO_2_ and RER. These 30- and 60-min responses were pooled, and one overall exercise session (60-min) value was calculated for the measurements. Subsequently, these pooled exercise values were used with standard non-protein RER-Energy Expenditure tables to calculate carbohydrate and fat substrate utilization and oxidation values [4,5].

### 2.2. Blood Procedures and Measures

Blood samples were centrifuged at 3000× *g* for 10 min (4 °C) to separate plasma (IEC Centra-8R centrifuge, International Equipment Company, Needham Heights, MA, USA). Plasma was transferred to cyro-freeze storage tubes and stored at −80 °C (Revco Corp., Cleveland OH, USA). Plasma estradiol-β-17 was measured using standard immunoassay techniques (Siemens Healthcare Technologies, Los Angeles, CA, USA; assay sensitivity 7.0 pmol/L). 

### 2.3. Data Analysis 

All data were analyzed using SPSS statistical software (version 25.0, Chicago, IL, USA). Descriptive statistics were determined for all measurements (means ± standard deviations (SD)). Normality of data distribution was assessed with the Kolmogorov–Smirnov test. The follicular vs. luteal pooled 60-min exercise responses for VO_2_, RER, and CHO-fat utilization and oxidation values as well as resting estradiol-β-17 were analyzed with paired *t*-tests. Effect sizes (Hedges’ g) were determined for all comparisons. Select relationships were examined with Pearson correlation analyses. Significance was set at α < 0.05. 

## 3. Results

Table 1 presents the physical characteristics and VO_2max_ values of the research participants. Based on the Cooper Clinic criteria our sample exceeded the 90th percentile for aerobic fitness relative to their VO_2max_ results [7].

Table 2 presents the VO_2_, CHO-fat utilization and oxidation values for the 60-min steady-state exercise sessions, as well as the resting estradiol-β-17. The VO_2_ responses did not differ between the two sessions (*p* > 0.05) and elicited 64.6 ± 7.2% and 64.5 ± 2.3% of VO_2max_ in the follicular and luteal sessions, respectively. 

CHO utilization and oxidation values in the luteal phase exercise sessions were significantly (*p* < 0.05) lower than in the follicular phase sessions. Conversely, fat utilization and oxidation values in the luteal phase exercise sessions were significantly (*p* < 0.05) greater than in the follicular phase sessions. The effect sizes for these findings (Table 2) were at the level of small to moderate. Additionally, there was a significantly greater concentration of resting estradiol-β-17 at the luteal session compared to the follicular session (*p* < 0.01).

For the correlational analyses, delta values were calculated (Δ = luteal session response—follicular session response) for the following variables: estradiol-β-17, fat—CHO oxidation (oxidation was deemed most physiologically relevant). The delta values were then correlated to one another. Delta estradiol-β-17 was significantly positively correlated with delta fat oxidation (i.e., as estradiol-β-17 increased from follicular to luteal, fat oxidation during exercise also increased (r = +0.62, *p* < 0.01)). Delta estradiol-β-17 was also significantly correlated with delta CHO oxidation, but in a negative manner (i.e., as estradiol-β-17 increased from follicular to luteal, CHO oxidation during exercise decreased (r = −0.52, *p* < 0.01)).

## 4. Discussion

The purpose of this study was to examine the role, if any, of estradiol-β-17 changes across the menstrual cycle on the energy substrate utilization and oxidation in eumenorrheic adult women during aerobic exercise. Our key finding was a greater usage in the amount of fat and a reduced amount of CHO in the luteal compared to follicular exercise sessions. Furthermore, the changes in fat and CHO oxidation from the follicular to luteal phase were directly related to the change in resting estradiol-β-17 concentrations. These findings agree with that of previous studies [8,9]. Furthermore, the findings support the supposition by Bunt in her landmark 1990 article on women, estrogens, and exercise, that estrogen can influence exercise metabolism by increasing lipid utilization, from which we based our hypothesis [3]. While our findings are not novel, they represent one of the largest sample-size-based studies to substantiate these outcomes and overcome the constraints of many previously published works (i.e., limited in statistical power due to too few participants).

Research has linked estrogens to increased lipolysis via direct and indirect physiological mechanisms for several decades [3,10,11]. Specifically, Newell-Fugate reports the effects are due to enhanced lipolytic enzymatic activity [12]. However, this is not a universally accepted finding, and some studies are equivocal on this point [1,13]. This entire line of research is hampered by the lack of scientific rigor and uniformity in some studies as to the approach of studying sex steroid hormonal changes across the menstrual cycle [13].

### Strengths and Limitations of the Study

Strengths of this investigation include the aforementioned sample size and the use of biochemical measures of estrogen concentration (estradiol-β-17) in conjunction with ovulation testing and self-report data as a means to assess the menstrual cycle phase. 

Our study also has several limitations that must be noted. First, our substrate metabolism calculations are based on non-protein respiratory exchange ratio values. Measuring and calculating the protein contributions to exercise energy metabolism would strengthen our conclusions. However, we felt the level of subject burden for the nitrogen collection procedure to assess protein was too excessive. Furthermore, the work of Lemon and associates suggests for the type of exercise our participants performed, the protein contributions would only be ~1% [14]. Secondly, our findings are based entirely on respiratory gases and there were no supportive biochemical biomarkers to assess energy metabolism. In hindsight, this was an omission on our part and as such weakens our conclusions. Nonetheless, the gases were obtained when the participants were in a steady state and substantial evidence supports the efficacy of such gas measurements under such conditions to accurately represent an individual’s metabolic state [15]. 

## 5. Conclusions

Our findings support the physiological concept that changes in estradiol-β-17 across the menstrual cycle are associated with changes in energy substrate utilization-oxidation in physically active women. Whether these changes have impacts on athletic performance in women remains to be determined. Future research should pursue this question as it has important implications for exercising females.

## Figures and Tables

**Table 1:** Physical characteristics of the study participants (mean ± SD).

Measure	Mean	Standard Deviation
Age (y)	22.4	3.8
Height (cm)	165.6	6.4
Weight (kg)	61.8	7.8
BMI (kg/m^2^) VO_2max_ (mL/kg/min)	22.5 47.9	2.5 12.0

**Table 2:** Metabolic responses for the exercise sessions in the follicular and luteal phases of the menstrual cycle.

Measure	Mean ± SD	Probability	Effect Size
Estradiol-β-17 (pmol/L) Follicular Luteal			
	243.8 ± 143.2		
	518.5 ± 285.4	<0.01	0.75
VO_2_ (L/min) Follicular Luteal	1.89 ± 0.48		
	1.90 ± 0.50	>0.05	0.21
CHO Utilization (%) Follicular Luteal			
	58.2 ± 15.1		
	51.6 ± 16.7	<0.05	0.45
CHO Oxidation (g/min) Follicular Luteal	1.38 ± 0.60		
	1.22 ± 0.56	<0.05	0.27
Fat Utilization (%) Follicular Luteal	41.8 ± 15.1 48.4 ± 16.7	<0.05	0.45
Fat Oxidation (g/min) Follicular Luteal	0.41 ± 0.14 0.49 ± 0.19	<0.05	0.28

## Data Availability

These data are available via contacting the senior author.

## References

[1] Mujika I., Taipale R.S. (2019). Sport science on women, women in sport science. Int. J. Sports Physiol. Perform..

[2] Lebrun C.M., Rumball J.S. (2001). Relationship between athletic performance and menstrual cycle. Curr. Womens Health Rep..

[3] Bunt J.C. (1990). Metabolic actions of estradiol: Significance for acute and chronic exercise responses. Med. Sci. Sports Exerc..

[4] Pollock M.L., Wilmore J.H. (1990). Exercise in Health and Disease: Evaluation and Prescription for Prevention and Rehabilitation.

[5] Glass S., Dwyer G. (2007). ACSM’s Metabolic Calculations Handbook.

[6] Allen A.M., McRae-Clark A.L., Carlson S., Saladin M.E., Gray K.M., Wetherington C.L., McKee S.A., Allen S.S. (2016). Determining menstrual phase in human biobehavioral research: A review with recommendations. Exp. Clin. Psychopharmacol..

[7] Pescatello L.S., Arena R., Riebe D., Thompson P.D., American College of Sports Medicine (2014). Health-related physical fitness testing and interpretation. ACSM’s Guidelines for Exercise Testing and Prescription.

[8] Nicklas B.J., Hackney A.C., Sharp R.L. (1989). The menstrual cycle and exercise: Performance, muscle glycogen, and substrate responses. Int. J. Sports Med..

[9] Hackney A.C., McCracken-Compton M.A., Ainsworth B. (1994). Substrate responses to submaximal exercise in the midfollicular and midluteal phases of the menstrual cycle. Int. J. Sport Nutr..

[10] Hansen F.M., Fahmy H., Nielsen J.H. (1980). The influence of sexual hormones on lypogenesis and lipolysis in rat fat cells. Acta Endocrinol..

[11] Jurkowski J.E., Jones N.L., Toews C.J., Sutton J.R. (1981). Effects of menstrual cycle on blood lactate, O_2_ delivery and performance during exercise. J. Appl. Physiol..

[12] Newell-Fugate A.E. (2017). The role of sex steroids in white adipose tissue adipocyte function. Reproduction.

[13] Elliott-Sale K.J., Minahan C.L., de Jonge X.A.K.J., Ackerman K.E., Sipilä S., Constantini N.W., Lebrun C.M., Hackney A.C. (2021). Methodological considerations for studies in sport and exercise science with women as participants: A working guide for standards of practice for research on women. Sports Med..

[14] Lemon P.W., Yarasheski K.E., Dolny D.G. (1984). The importance of protein for athletes. Sports Med..

[15] Romijn J.A., Coyle E.F., Hibbert J., Wolfe R.R. (1992). Comparison of indirect calorimetry and a new breath 13C/12C ratio method during strenuous exercise. Am. J. Physiol..

